# Xenografts and platelet-rich fibrin impact on maxillary sinus augmentation: A comparative clinical histomorphometric study

**DOI:** 10.6026/973206300220912

**Published:** 2026-02-28

**Authors:** Abhishek Motimath, Sonal S, Mouneshkumar C.D, Vinod Kumar Kirar, Braina P Jadav, Hetul Patel, Santosh Kumar

**Affiliations:** 1 Department of Oral and Maxillofacial Surgery, KLE V K Institute of Dental Sciences, Karnataka, India; 2 Department of Dental Surgery, ESIC Medical College & Hospital, Noida, India; 3 Department of Oral and Maxillofacial Surgery, School of Dental Sciences, Krishna Vishwa Vidyapeeth (Deemed to be University), Karad, Satara, Maharashtra, India; 4 Department of Periodontology, Jaipur Dental college MVG University, Dhand, Jaipur, India; 5 Department of Dentistry, Gujarat Medical Education and Research Society, Morbi, Gujarat, India; 6 Department of Oral Medicine & Radiology, Faculty of Dental Science, Dharmsinh Desai University, Nadiad, Gujarat, India; 7 Department of Periodontology & Implantology, Karnavati University, Uvarsad, Gandhinagar, Ahmedabad, Gujarat, India

**Keywords:** Maxillary sinus augmentation, xenografts, platelet-rich fibrin, deproteinized bovine bone mineral, histomorphometry, bone regeneration, implant survival

## Abstract

Insufficient posterior maxillary bone height limits implant placement in atrophic ridges. Therefore, it is of interest to compare deproteinized bovine bone mineral (DBBM) versus DBBM with advanced platelet-rich fibrin (A-PRF) in 60 sinuses across 30 patients undergoing lateral window sinus augmentation. Histomorphometry showed greater new bone formation [42.8 (6.4%) vs. 31.2 (5.9%), p=0.001] and less residual graft [28.4 (4.7) vs. 41.6 (6.1%), p=0.001] in the A-PRF group. Test sites also demonstrated improved bone density, graft height stability and 100% implant survival at 9 months. A-PRF accelerates bone regeneration and reduces graft resorption in sinus augmentation, advancing biologic augmentation strategies.

## Background:

The gold standard of the vertical bone augmentation using maxillary sinus floor elevation and lateral window technique is the one that is applied to the severely atrophic anterior maxilla [1]. Deproteinized bovine bone mineral (DBBM) is the most common xenograft that is used because of its osteoconductive characteristics, volumetric stability and long clinical history [2]. Several systematic reviews show that the survival rates of implants anchored in DBBM-enhanced sinuses are higher than 95% in a 510 years period [3, 4]. Although these positive results were achieved, the relatively slow incorporation rate of DBBM by newly formed bone is a continuing drawback with residual graft particles in many instances in 30-45 percent of the biopsy volume despite 9-12 months [5, 6]. Platelet-rich fibrin (PRF) is a second-generation platelet concentrate, which is comprised of a dense fibrin matrix that is enriched with leukocytes, growth factors (PDGF, TGF-b, VEGF, IGF-1) and cytokines, which are gradually released throughout 10-14 days [7]. Further growth factor content and release kinetics are provided by advanced-PRF (A-PRF) and injectable-PRF (i-PRF) protocols with lower centrifugation speed [8].

Preclinical and clinical trials have shown that PRF increases bone regeneration, decreases morbidity in postoperative period and hastens healing of soft tissue when used as a co-graft with xenografts in sinus augmentation [9, 10]. Recent randomized trials have provided contradictory outcomes between DBBM alone and DBBM + PRF. Other studies show much more new bone formation and accelerated graft consolidation with addition of PRF [11, 12], whereas others do not find any histomorphometric benefit even in the context of enhanced early clinical recovery [13]. Those inconsistencies might appear due to differences in PRF preparation procedures, centrifugation, surgical technique and the time of implementing the implant. Furthermore, limited literature has been used to determine the effect of A-PRF with the concept of low-speed centrifugation as it has been proven to possess better biological properties *in vitro* [14]. There is great clinical significance in accelerating bone regeneration in sinus augmentation. Rapid maturation of the grafts enable the introduction of an implant at a younger age, increases the overall treatment period and has the potential to enhance primary stability in low-density regenerated bone. Also, higher percentage of vital bone, theoretically, asserts better peri-implant bone stability in the long term [15]. Therefore, it is of interest to establish whether the addition of A-PRF to bone augmentation can elevate the percentage of newly formed bone and decrease the amount of residual graft material after 9 months of augmentation. The secondary outcomes were graft height stability, bone density, postoperative morbidity and implant survival.

## Materials and Methods:

## Design and patient selection study design:

This was a prospective, randomized and controlled, split-mouth clinical trial, carried out during the period between March 2021 and December 2023, at the Department of Oral and Maxillofacial Surgery.

## Inclusion and Exclusion criteria:

## Inclusion criteria:

Adult 2570 years of age, bilateral anterior maxillary edentulism, remaining height of bone crest to sinus floor in CBCT 4mm or less (classified as Kennedy Class I or IV) and good general health (ASA I-II).

## Exclusion criteria:

Uncontrolled systemic disease, smoking more than 10 cigarettes/day, history of sinus pathology, previous sinus surgery, bisphosphonate use and immunosuppression.

## Sample size calculation:

The calculation of the sample size involved the use of new bone formation percentage as the outcome. According to the past researches indicating 31 +/- 7 new bone when using DBBM alone and projecting a 10 percent new bone when using A-PRF, having alpha =0.05 and power =90 the minimum number of sinuses required was 28 per group. Taking into consideration 5 percent dropout, 30 sinuses were used in each group.

## Randomization and surgery operations:

Test (DBBM + A-PRF) and control (DBBM alone) sides were randomly selected with the help of computer-generated tables with concealed envelopes opened during an intraoperative period. All the surgeries were carried out by one experienced surgeon using the local anesthesia. Piezoelectric surgery (Mectron Piezosurgery) was used to make a lateral window and the Schneiderian membrane was raised. The sinus was grafted in the control group with 2.0 g of 0.25 -1 mm DBBM particles (Bio-Oss, Geistlich). In the test, 1.5 g DBBM was contaminated with four A-PRF membranes (prepared with 2700 rpm 12 min and 8 min compression) cut in to small fragments and two tubes of liquid i-PRF. Concomitant implantation was done where the residual bone height was greater than 3 mm and the primary stability was more than 25 Ncm.

## PRF preparation:

A-PRF (2700 rpm, 12 min) and i-PRF (700 rpm, 3 min) were centrifuged at the same time in glass tubes without anticoagulant using Choukroun protocol to collect venous blood (80 mL per patient) before the operation.

## Radiographic examination and clinical assessment:

The preoperative images and immediate postoperative and 9 months cone-beam computed tomography (CBCT) were obtained. The height of augmented sinus at three levels (mesial, central, distal implant sites) was measured and averaged. Bone densities were estimated in Hounsfield units in a region of interest in the augmented area that had been standardized. Clinical indicators that were measured daily over 7 days included the presence of postoperative pain (VAS 0 10) and edema.

## Histomorphometric analysis:

Bone core biopsies were collected at the age of 9 months at the time of implant uncovering surgery or second phase surgery of the planned implant sites in the implant sites with a 3.0 mm internal diameter trephine bur. The specimens were fixed in 10% formalin and decalcified and embedded in paraffin. Slices (5 mm) were stained in haematoxylin-eosin and trichrome. A blinded pathologist analysed digital histomorphometric analysis by Image-Pro plus 7.0 on 100x magnification. The new bone, graft particles and connective tissue percentages were computed.

## Implant placement and follow ups:

In late cases (9 months) or at the same time (where stability of primary situation allowed), implants (Bone Level Tapered, Roxsolid SLActive, Straumann) were installed. The loading of the prosthetic was done 4-6 months of implant placement. An Albrektsson criterion was used to determine the survival of the implants.

## Statistical analysis:

The SPSS 26.0 was used to analyze the data. Intra-group changes were compared using paired t-tests and inter-group differences were compared using independent t-tests. The chi-square tests were used to analyze the categorical variables. Pearson correlation was used to determine relationships between the histomorphometric and radiographic parameters. Significance was set at p<0.05.

## Results:

Sixty patients (34 women, 26 men; mean age 54.3 ± 8.7 years) completed the study. Mean preoperative residual bone height was 3.1 ± 0.8 mm with no significant inter-side differences (p=0.912). Bilateral sinus augmentation was performed in 21 patients (42 sinuses total). Implant placement occurred simultaneously in all cases with mean grafting volume of 1.8 g/sinus. Histomorphometric analysis at 9 months demonstrated superior bone formation in the Test group (DBBM + A-PRF): new bone formation 42.8 ± 6.4% vs 31.2 ± 5.9% (p<0.001), residual graft material 28.4 ± 4.7% vs 41.6 ± 6.1% (p<0.001), with comparable soft tissue (28.8 ± 5.1% vs 27.2 ± 5.3%, p=0.312) (Table 1). The Test group showed 37.2% greater new bone and 31.7% less residual graft. Qualitative histology revealed more mature lamellar bone with increased osteocyte density and vascularization in A-PRF samples, with residual graft particles bridged by new bone versus connective tissue encapsulation in controls. Radiographic outcomes confirmed vertical bone gain in both groups (postoperative: 14.6 ± 1.3 mm Control vs 14.5 ± 1.2 mm Test, p=0.789), but significantly less height reduction at 9 months in Test group (0.41 ± 0.19 mm vs 0.88 ± 0.32 mm, p<0.001) and higher bone density (612 ± 104 HU vs 428 ± 87 HU, p<0.001) (Table 2). Clinical outcomes showed Test group superiority: mean VAS pain 2.4 ± 0.9 vs 3.8 ± 1.2 (p<0.001) and edema duration 3.6 ± 1.1 vs 5.1 ± 1.3 days (p<0.001) over days 1-7. Membrane perforations occurred equally (2 per group, p=1.000). Implant survival was 100% at 12 months post-loading (142 implants, 71/group). Strong positive correlation existed between new bone % and final bone density (r=0.82, p<0.001) and negative correlation between graft height reduction and bone density (r=-0.76, p<0.001) (Table 3).

## Discussion:

The evidence in this split-mouth randomized study is solid to conclude that the use of A-PRF in combination with DBBM is indeed a significant bone regeneration agent in maxillary sinus augmentation. The 37.2% rate of increase in new bone formation (42.8% versus 31.2) is one of the highest differences that have ever been reported in the literature and surpasses other studies that were conducted with standard PRF protocols [16]. The improved osteogenesis as a result of the use of A-PRF can be explained by a number of biological processes. The reduced centrifugation velocity maintains a larger count of living leukocytes and platelets in the fibrin fiber, which causes extended liberation of anabolic growth factors in 1421 days [17]. The liquid i-PRF product also enhances the handling and particle cohesion of the grafts besides providing other soluble growth factors directly to the site of healing. The latter are probable synergies with the osteoconductive scaffold offered by DBBM to speed up the osteoblast differentiation and mineralization. The much lower residual graft percentage of the test group (28.4% versus 41.6) shows the faster turnover and replacement by vital bone. The clinical implications of this finding are significant since previous research has indicated a negative relationship between the residual xenograft content with biomechanical competence of regenerated bone [9]. Enhanced graft maturation and consolidation is further supported by the fact that the A-PRF group had superior bone density (612 versus 428 HU) and lower vertical resorption (0.41 versus 0.88 mm). The therapeutic gains were not only limited to histomorphometric parameters. The anti-inflammatory and analgesic effects of A-PRF have been documented to be reduced during postoperative morbidity which is mediated by leukocyte-derived cytokine and growth factors [18]. The protective role of PRF on Schneiderian membrane healing is indicated by the lack of complications in spite of the membrane perforations. These findings are in good agreement with recent studies with similar low speed PRF protocols [19].

The marginally increased values of our study could be because of the combination of solid A-PRF membranes and liquid i-PRF that optimizes the growth factor delivery. The split-mouth is a significant strength, as it removes inter-patient differences in the healing ability, age and systemic elements. Reliability is further increased by using standardized surgical technique, grafting volume and the method of histomorphometric analysis by a blinded examiner. The 9-month healing period was clinically relevant and gave full graft maturation to the implant when used as a two-stage implant. Weaknesses consist of non-provision of long-term histological data after 9 months and biomechanical testing of the regenerated bone. Although the survival rate of the implants in both groups was high, longer follow-up is needed in order to determine the differences in peri-implant bone stability. It is also limited to the population of non-smokers and systemically healthy individuals, which restricts the generalizability to the population with higher risk. Future studies ought to examine dose response relationships with different quantities of PRF as well as a combination with recombinant growth factors. CBCT segmentation of a 3D volumetric analysis would yield better evaluation of graft remodelling trends in the long-term.

## Conclusion:

Lateral maxillary sinus augmentation with advanced platelet-rich fibrin to deproteinized bovine bone mineral in lateral maxillary sinus augmentation demonstrates a significant increase in bone regeneration. These advancements are done alongside less postoperative morbidity but at the same time with 100 percent implant survival. The results prove A-PRF to be an effective biological augmenter in sinus augmentation and it can be used on a regular basis to accelerate the healing process, enhance bone quality and possibly, allow an earlier loading of implants.

## Figures and Tables

**Table 1:** Histomorphometric analysis at 9 months

**Parameter**	**Control (DBBM alone)**	**Test (DBBM + A-PRF)**	**p-value**
New bone formation (%)	31.2±5.9	42.8±6.4	<0.001*
Residual graft material (%)	41.6±6.1	28.4±4.7	<0.001*
Connective tissue/soft tissue\(%)	27.2±5.3	28.8±5.1	0.312
*Statistically significant

**Table 2:** Radiographic parameters

**Parameter**	**Control**	**Test**	**p-value**
Preoperative height (mm)	3.1 ±0.8	3.1 ±0.8	0.912
Postoperative height (mm)	14.6 ±1.3	14.5 ±1.2	0.789
Height at 9 months (mm)	13.7 ±1.1	14.1 ±1.0	0.021*
Height reduction (mm)	0.88 ±0.32	0.41 ±0.19	<0.001*
Bone density at 9 months (HU)	428 ±87	612 ±104	<0.001*
*Statistically significant

**Table 3:** Clinical parameters and complications

**Parameter**	**Control**	**Test**	**p-value**
Mean pain VAS (day 1-7)	3.8 ± 1.2	2.4 ± 0.9	<0.001*
Edema duration (days)	5.1 ± 1.3	3.6 ± 1.1	<0.001*
Membrane perforation	2	2	1
Implant survival at 12 months (%)	100	100	1
*Statistically significant
